# Midpalatal suture maturation stage assessment in adolescents and young adults using cone-beam computed tomography

**DOI:** 10.1186/s40510-019-0291-z

**Published:** 2019-10-08

**Authors:** Ludy Marileidy Jimenez-Valdivia, Violeta Malpartida-Carrillo, Yalil Augusto Rodríguez-Cárdenas, Heraldo Luis Dias-Da Silveira, Luis Ernesto Arriola-Guillén

**Affiliations:** 1 Division of Orthodontics, School of Dentistry, Universidad Científica del Sur, Lima, Peru; 2 Division of Oral Implantology, School of Dentistry, Universidad Científica del Sur, Lima, Peru; 3 Division of Oral and Maxillofacial Radiology, Faculty of Dentistry, Universidad Nacional de Colombia, Bogotá D. C, Colombia; 4 Division of Oral Radiology, Faculty of Dentistry, Federal University of Rio Grande do Sul, Porto Alegre, Brazil; 5 Division of Orthodontics and Division of Oral and Maxillofacial Radiology, School of Dentistry, Universidad Científica del Sur, Lima, Peru

**Keywords:** Midpalatal suture maturation stages, Palatal expansion technique, Cone-beam computed tomography

## Abstract

**Background:**

The aim of this study was to evaluate the midpalatal suture maturation stages in adolescents and young adults using cone-beam computed tomography (CBCT).

**Methods:**

The sample comprised 200 CBCT scans of individuals aged 10 to 25 years old (95 males and 105 females) divided into three groups, adolescents (*n* = 48), post-adolescents (*n* = 52), and young adults (*n* = 100). The Planmeca ProMax 3D software was used for the midpalatal suture maturation stage evaluation according to Angieleri’s method, using cross-sectional axial slice. Two previously calibrated examiners analyzed the images and classified according to five different maturation stages. A, B, and C stages were considered with open midpalatal suture, and D and E were considered without open midpalatal suture. Association tests were performed using chi-square test also, and a binary logistic regression was evaluated (*P* < 0.05).

**Results:**

The possibility to find open midpalatal suture in individuals of 10 to 15 years old was 70.8%, in subject aged 16 to 20 and 21 to 25 years old was 21.2% and 17%, respectively. Furthermore, this possibility in individuals older than 16 years was greater in males than in females.

**Conclusions:**

The possibility to find open midpalatal suture in post-adolescents and young adults is greater than the orthodontists considered years ago. Furthermore, men are more likely to find midpalatal suture opening. These implications might be considered by the orthodontists when maxillary expansion is required. Besides, the ossification of the middle palatal suture is very variable, and therefore, the use of CBCT might be recommended to clarify this possibility.

## Background

Transverse maxillary constriction is associated with several problems that include posterior crossbite (dental and/or skeletal), dental crowding, occlusal disharmony, pharyngeal airway narrowing, alterations in tongue posture, and mouth breathing, producing meaningful effects in muscular function and esthetic [1–3].

Rapid maxillary expansion (RME) is an orthopedic procedure that requires heavy forces to promote the separation of the midpalatal suture (MPS). This procedure leads to stretching of collagenous fibers as well as the local formation of a new bone that corrects the transverse maxillary constriction with a real increase in the transversal width [4]. Over the years, this technique has become a routine procedure in orthodontic treatment for patients who have a MPS opening. On the other hand, in patients with a full MPS ossification, the surgically assisted rapid maxillary expansion (SARME) has been recommended to reduce the resistance to the disjunction [5, 6]. However, the approximated age limit to shift from RME to SARME is not clear enough especially in late adolescents and young adults [7, 8]. Although several studies suggest that RME should be recommended before puberty [9, 10]. There are other reports of necropsy specimens that shown patients to have no signs of fusion of this suture at ages 27, 32, 54, and even 71 years [11–13]. Thus, the chronological age is an unreliable parameter for evaluating the developmental status of the MPS during growth [13, 14].

In 2013, Angelieri et al. [15] proposed a method of individual evaluation of MPS maturation using cone-beam computed tomography (CBCT). These authors determined five maturational stages (A, B, C, D, and E) as a way of providing more reliable clinical data when making the decision between RME only and SARME for adolescent and young adult patients. According to this, patients in stages A and B would have less resistance and greater skeletal effects of RME than in stage C. Meanwhile, for patients in stages D and E, SARME was recommended. Three years later, Angelieri et al. [16] confirmed that in spite of increased sutural resistance to conventional RME at stage C, the widening of maxilla orthopedically with no surgical interventional still is possible. Recently, Tonello et al. [17] evaluated the maturation stage of the MPS using CBCT images in Brazilian adolescents from 11 to 15 years old using the same Angelieri’s method. These authors reported that stage C was the most prevalent without differences in the maturation stages between boys and girls in this specific age group. In this way, Ladewing et al. [18] evaluated the maturational stage in post-adolescents from 16 to 20 years old using the same method and they concluded that stages C, D, and We were the most representative and shown that both sexes had a higher prevalence of stage C. These results showed that great variability in the stages of calcification of the middle palatal suture could occur regarding the possibility to make a maxillary disjunction and the chronological age. Moreover, these results may change according to the racial group.

The literature reports that skeletal maturity is usually reached earlier in girls than in boys in the pubertal ages [19, 20]. Likewise, this was confirmed when a MPS was used as a maturational tool, despite a statistically significant difference was not observed [16–18]. However, to the best of our knowledge, there is a lack of evidence regarding the age group limits and the RME possibility in different populations and it is helpful for the clinical practice. Therefore, the aim of this study was to evaluate the midpalatal suture maturation stages in an urban sample of adolescents, post-adolescents and young adults associated with chronological age and sex assessing MPS maturational stages by using CBCT scans. The knowledge of the percentage on the possibility to find midpalatal suture opening in young adults at first will help the orthodontist to offer the patient a possible treatment plan that will be corroborated with an auxiliary examination. Furthermore, the clinicians may know about the probability to perform RME in post-adolescents and young adults.

## Methods

This descriptive and retrospective study was approved by the Ethics Committee of Universidad Cientifica del Sur, Lima, Perú, with the approval number 159-2018-POS8. The sample consisted of 200 CBCT scans of adolescents (*n* = 48), post-adolescents (*n* = 52), and young adults (*n* = 100) aged between 10 to 25 years (95 males and 105 females, Table 1) attended in a private dental diagnostic imaging center (Lima, Perú). All patients signed the donation form.

**Table 1:** Distribution of the MPS maturational stages by age group and sex

Age (years)	Sex	Stage										Total
		A		B		C		D		E		
		*n*	%	*n*	%	*n*	%	*n*	%	*n*	%	
10–15	F	1	3.2	6	19.4	14	45.2	7	22.6	3	9.7	31
	M	1	5.9	7	41.2	6	35.3	2	11.8	1	5.9	17
	F + M	2	9.1	13	60.6	20	80.5	9	34.4	4	15.6	48
16–20	F	0	0	1	4.8	3	14.3	8	38.1	9	42.9	21
	M	0	0	0	0	7	22.6	13	41.9	11	35.5	31
	F + M	0	0	1	4.8	10	36.9	21	80	20	78.4	52
21–25	F	0	0	0	0	5	9.4	16	30.2	32	60.4	53
	M	0	0	2	4.3	10	21.3	12	25.5	23	48.9	47
	F + M	0	0	2	4.3	15	30.7	28	55.7	55	109.3	100
TOTAL		2	1	16	8	45	22.5	58	29	79	39.5	200

*F* female, *M* male

The sample size was determined by applying the formula to estimate one proportion (possibility to find midpalatal suture opening in individuals older than 18 years) with a 95% confidence level, the precision of 5% and 10% proportion of this possibility (data from a previous pilot test). The minimum required sample was 163 CBCTs.

Based on the inclusion criteria, CBCT scans were selected from patients aged 10 to 25 years, both sexes who underwent CBCT imaging for the diagnosis of skeletal malocclusion and impacted teeth from January 2017 to December 2018. The exclusion criteria were subjects with previous orthodontic treatment or any appliance, maxillofacial trauma, odontogenic pathologies, cleft lip and palate, syndromic conditions, and the presence of noise or blurred images on the CBCT scans.

All CBCT images evaluated in the current study were obtained using a Planmeca ProMax 3D Mid scanner (Helsinki, Finland) adjusted to the following specifications: a field of view of at least 11 cm, 90 kV, 10 mA, a voxel size of 0.2 to 0.3 mm, and exposure time of 13.68 s. CBCT images were analyzed using Planmeca Romexis**®**. The adjustment of the patient’s head in the three planes of space and the selection of the slice for evaluation of the MPS maturational stages were performed according to the protocol described previously [15].

**Fig. 1:**
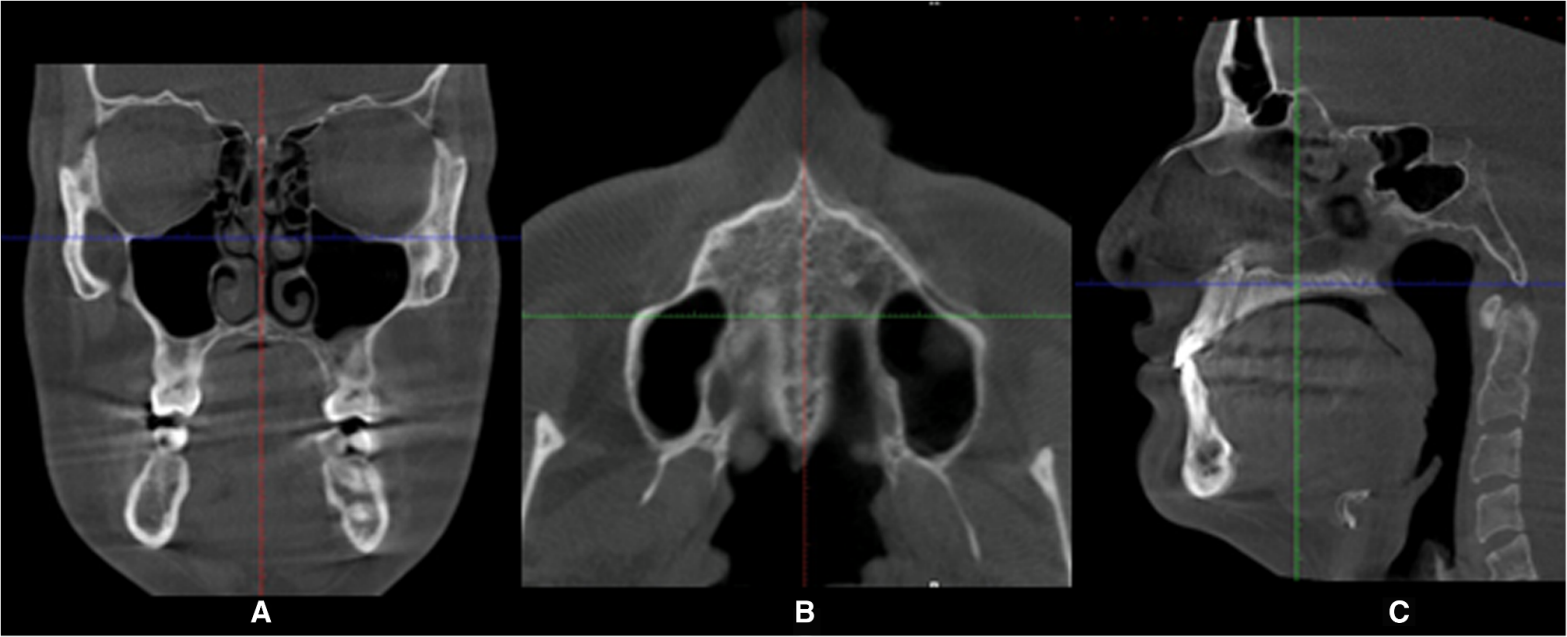
Procedures in the CBTC sections to measure the midpalatal suture. **a** Coronal view. **b** Axial view. **c** Sagittal view, note in the view that the blue line is positioned through the center of the hard palate

The images were obtained in a standardized way. First, in the coronal (Fig. 1a) and axial (Fig. 1b) views, the cursor of the image analysis software was positioned at the patient’s midsagittal plane. Afterward, in the sagittal view, the patient’s head was adjusted so that the horizontal reference line coincided with the median region of the palate, which is the cancellous bone between the upper and lower cortical bones (Fig. 1c).

**Fig. 2:**
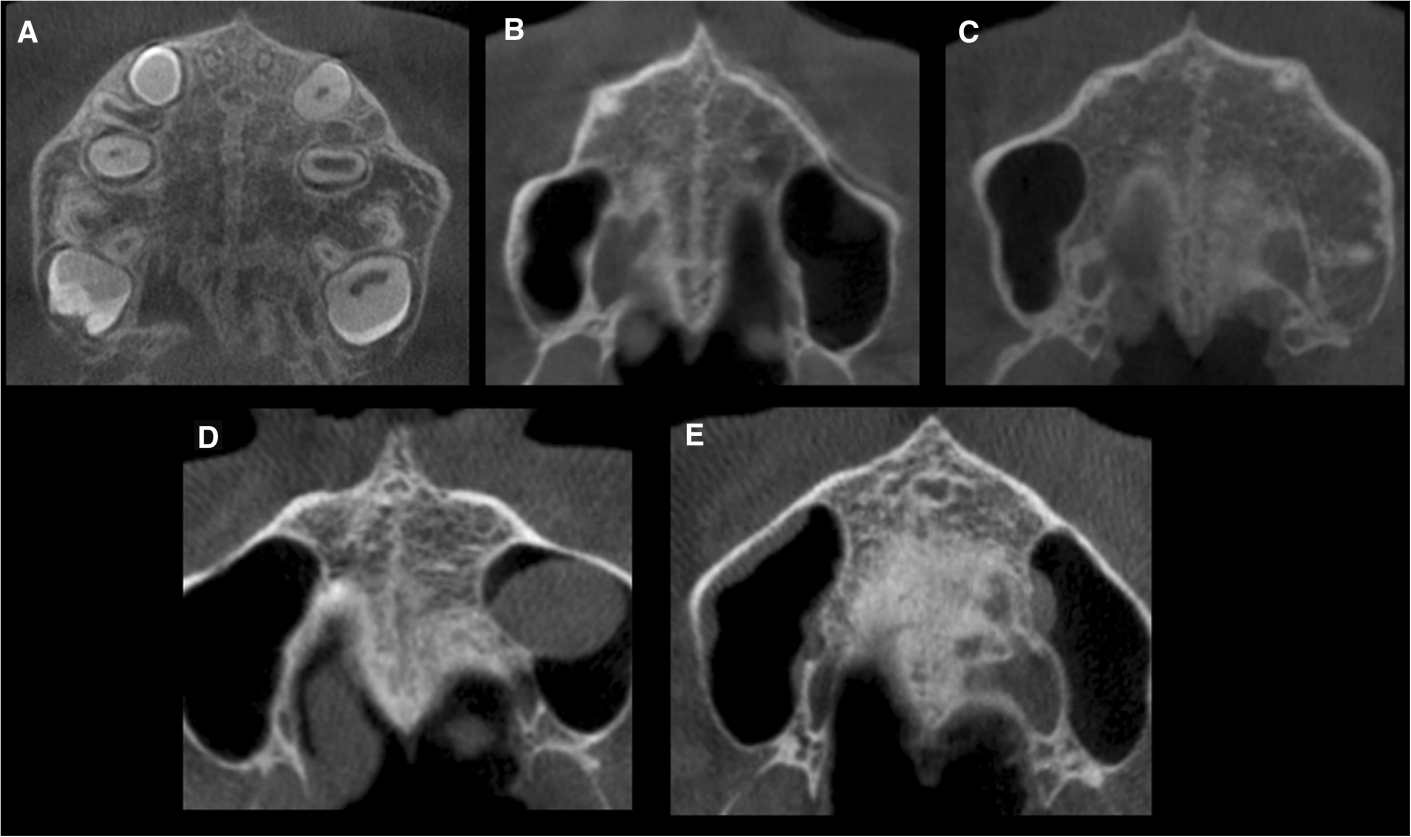
Method of Angieleri et al. [15] in CBCT. **a** The midpalatal suture is seen as a relatively straight radiopaque line. **b** The midpalatal suture appears as a scalloped line of high density. **c** Two radiopaque, scalloped, and parallel lines are separated by areas of low radiographic density. **d** The palatine bones become more radiopaque, and the suture is not visualized in this sector only is visualized as two scalloped high-density lines at the midline on the palate bone. **e** It is no longer possible to see the suture along the maxillary and palatine bones, indicating fusion l; fusion has occurred in the maxilla

Subsequently, in the axial CBCT section, the visualization and classification of the skeletal maturation stage of the MPS were conducted according to the method of Angelieri et al. [15]. For a more precise evaluation, two axial cross-sectional slices were used when subjects presented with a thick or a curve palate, according to the previous recommendations [15, 17, 18]. Two previously calibrated examiners analyzed the images and classified according to five different maturation stages. A, B, and C stages were considered with open midpalatal suture, and D and E were considered without open midpalatal suture (Fig. 2).

The training and calibration were conducted by an experienced and trained orthodontist (LEAG) using 50 CBCTs slices of adolescents and post-adolescents of both genders aged 10–25 years randomly selected. The observer (LMJV) received a detailed explanation of the morphologic features of each MPS maturation stage in a PowerPoint (Microsoft, Redmond, Wash, USA) high-resolution image presentation containing 50 CBCT axial slices. The images were identified only by codes and the observer was trained in the maturation stage of the MPS and for classify the MPS method using exact figures and legends described by Angelieri et al. [15]. Furthermore, the observer received a hard-copy handout with a written description of the radiographic features of each MPS maturation stage.

The calibration was done twice with a washout period of 4 weeks. Recorded data were submitted to the agreement analysis to check inter-examiner errors. In addition, the observer was asked to re-observe the slices to rule out any intra-examiner error. The weighted kappa coefficient was used for both analyses. The 200 CBCT sagittal slices were observed in a dimly lit room with constant light intensity. Four weeks after the first observation, the examiner was retrained in the maturation stage of the MPS receiving the images in a different random order, and she was asked to re-observe the slices.

### Statistical analyses

The weighted kappa coefficients were calculated for evaluation of the intra- and inter-examiner measurement error using the STATA version 16 (Stata Corp., College Station, TX, USA), and the results were interpreted according to the scale of Landis and Koch [21]. All statistical procedures were conducted with SPSS version 24 (SPSS Inc., Armonk, NY, USA) software for windows.

The chi-square test was used to analyze the possibility to find open midpalatal suture by age groups. Finally, a binary logistic regression model was performed using the maturation stage of the MPS as an outcome variable. The predictor variables were age (in years) and sex (the codes were 0 and 1 for females and males, respectively). The impact of each factor on the outcome variable was expressed as an OR with its 95% confidence interval (95% CI). Statistical significance for all statistical tests was set at *P* < 0.05.

## Results

The weighted kappa coefficients for the evaluation of the intra- and inter-examiner measurement error in the MPS maturation stage were 0.89 and 0.90, respectively, demonstrating almost perfect agreement according to the scale of Landis and Koch [21].

The most frequent maturation stage in the study population (Table 1) was stage E (39.5%), followed by stages D (29%), C (22.5%), B (8%), and A (1%). The MPS was not fused in 63 out of 200 subjects (31.5% of the total sample with stages A, B or C). In the females, there were higher prevalence of stage C (45.2%) in the age group od 10 to 15 years. The stage C decreased from 45.2% (14 subjects) in the younger group to 9.4% (5 subjects) in the older group. As expected, in the older age group (21–25 years), the frequencies of stages D and E were higher than in the 16- to 20-year age group. Still, in the group between 21 and 25 years, no subject was observed in stage A. However, the stage B was found in two subjects in the age group 21 to 25 years.

When considering all groups (Table 2), we observed that in the age group of 10 to 15 years is possible to verify open midpalatal suture in 70.8%, while in the age group 16 to 20 years and 21 to 25 years is possible to verify midpalatal suture opening in 21.2% and 17%, respectively. Furthermore, the findings show that in the age group 16 to 20 years and 21 to 25 years, males have more possibility to present midpalatal suture opening than females.

**Table 2:** Distribution of the MPS maturational stages by age group and sex regarding the possibility to find midpalatal suture opening

Midpalatal suture opening							*P*
Possibility				No possibility			
Age (years)	Sex	*n*	%	*n*	%	Total	
10–15	F	20	41.6	11	22.9	31	
	M	14	29.2	3	6.3	17	
	F + M	34	70.8	14	29.2	48	0.320
16–20	F	4	7.7	17	32.7	21	
	M	7	13.5	24	46.1	31	
	F + M	11	21.2	41	78.8	52	1.000
21–25	F	5	5.0	48	48.0	53	
	M	12	12.0	35	35.0	47	
	F + M	17	17.0	83	83.3	100	0.037^*^

*F* female, *M* male

^*^Chi-square test: *P* < 0.05, significant

The comparison of the maturation stages by sex is given in (Table 3). It shows that both sexes had a higher prevalence of stage E, which is more frequent in females (41.9%), followed by stage D (29.5%). Stage C was observed in 20.9%. The prevalence of stages A and B in females was 1% and 6.7% had the lowest prevalence. In males, stage E was the most prevalent (36.8%), followed by stage D (28.4). Stage C was observed in 24.2%, stage A in 1.1%, and stage B in 9.5%.

**Table 3:** Distribution and comparison of the MPS maturational stages in 10- to 25-year-old subjects by sex

Sex	Stage										Total
	A		B		C		D		E		
	*n*	%	*n*	%	*n*	%	*n*	%	*n*	%	
Female	1	1.0	7	6.7	22	20.9	31	29.5	44	41.9	105
Male	1	1.1	9	9.5	23	24.2	27	28.4	35	36.8	95

Chi-square test: *P* = 0.898, not significant

The results of logistic regression (Table 4) showed that the female sex has a 51.1% lower probability to find midpalatal suture opening. In terms of age, for each year that increases the age, there is a 24% lower probability to find midpalatal suture opening.

**Table 4:** Results of the logistic regression model with the maturational stages of the midpalatal suture as outcome variable and age and sex as predictors

Variable	*P*	OR	95% CI	
			Lower	Upper
Sex	0.049	0.489	0.239	0.998
Age (years)	< 0.001	0.760	0.695	0.831
Constant	< 0.001	111.891		

*r*^2^ Cox *y* Snell = 21%

*r*^2^ Nagelkerke = 30%

## Discussion

The treatment of transverse maxillary constriction in patients is an important topic for orthodontists, and this is especially challenge in late-stage adolescent and young adult patients because there is no consensus in the literature regarding the minimum age for reliable palatal expansion. Until a few years ago, the possibility to find midpalatal suture opening and therefore of performing a maxillary disjunction was considered only until 16 years old. In addition, attempting to choose RME in older individuals can lead to consequences such a significant pain, gingival recession, palatal mucosa ulceration or necrosis, buccal tipping of the posterior teeth, and reduction of buccal bone thickness [22–24] alveolar bone bending [25], buccal root resorption [26], and fenestration of the buccal cortex [27]. On the other hand, SARME implies possible unnecessary surgical procedures increasing morbidity, cost, risk, and more days required for patient recovery [28].

**Fig. 3:**
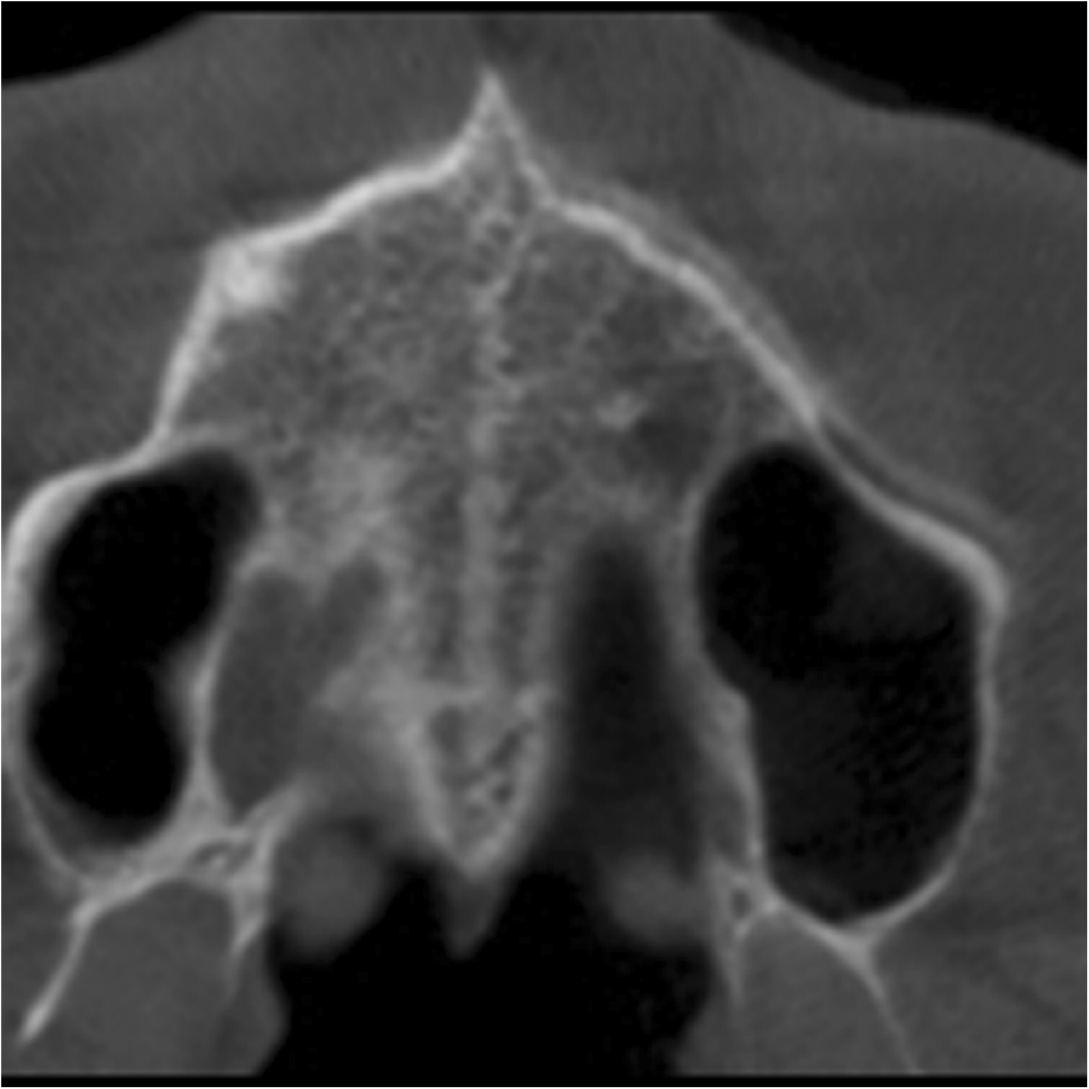
Example of the midpalatal suture condition in a patient of 25-year-old boy in stage B appears as a scalloped high-density line

More recent studies pointed out that in young adult patients, this possibility [11–13], in essence, is greater and suggests that it is possible to achieve RME until 20 years. In this way, with the advent of the miniscrew-assisted rapid palatal expansion technique (MARPE), cases of success have been reported in patients up to 24–26 years old [29–31]. However, there are few studies that determine this proportion until 25 years and this information is valuable for orthodontists who, comprehending this rate, may consider the RME as a possibility if the case merits and not only a dental expansion (Fig. 3).

Usually, the therapeutic decision to treat a posterior crossbite is supported by the chronological age of the patient, due to the scientific literature that sustains the realization of RME in growing patients [27]. However, there is no consensus regarding the decision of RME or SARME; some authors recommend SARME to patients older than 25 years or older [32], and other references as Epker and Wolford [6] recommend patients over 16 years. The great variability in the indications of age ranges and the absence of precise clinical guidelines regarding the treatment time for maxillary expansion were the reasons to perform this research. This study seeks to determine the possibility to find midpalatal suture opening to do maxillary disjunction in adolescent, post-adolescent, and young adult patients as a less invasive alternative to SARME.

To evaluate the morphology of the MPS according to the stages of maturation of Angieleri et al. [15], we measured CBCTs through a cross section in the middle of the palate. This method determines five maturation stages of the MPS. The A, B, and C stages were defined as possible to find midpalatal suture opening with more favorable results, being C a critical stage. According to a clinical trial about maxillary expansion, RME under D maturation stage is not feasible to the posterior region despite the interincisal opening in the maxillary bone portion, causing a failure of the RME procedure. Therefore, they suggest that in stages D and E, the treatment of surgically assisted RME would be better because the fusion of the suture has been partial or total, preventing the RME forces from opening the suture (Fig. 2).

Angieleri et al. [15] reported that stage A was observed in early childhood from 5 to 11 years of age, and stage B was observed mainly up to 13 years of age, similar results to our study, where stages A and B were observed in the range of 10–15 years old (Table 1). The fusion of the MPS was observed below 15 years, findings very similar to Angieleri et al. [16] who reported the fusion of the MPS in patients of 11 and 14 years. In the age group 10 to 15 years, stage C was found mainly in the females, which means that the maturation of the MPS occurs earlier in women than in men. Interestingly, these results corroborate the clinical findings of RME failure in late adolescents, mainly in females and adults.

Unlike the study of Ladewig et al. [18] where it shows that both sexes have a high prevalence of stage C, stages D and E were observed in our sample in 29% and 39.5%, respectively. Also, in our study, a high prevalence was found in the 21–25-year-old group, a similar result to previous studies, but with different results, they observed 84.4%, 60% in subjects older than 18 years who had stages D and E, respectively [16, 18]; this difference could be results of environmental and genetic characteristics of the sample evaluated. However, also, it means that in young adult patients, there may always be an opportunity for RME treatment [29–31].

Age and sex play an essential role to find midpalatal suture opening, but not crucial in the decision making because the literature reports that they are not reliable parameters to determine if the MPS is merged or not [11]. The possibility to find midpalatal suture opening is greater in the male sex compared to the female sex (Table 2). In addition, Capelozza Filho et al. [23] report an 80% success in RME in patients without growth. The RME was considered successful due to the creation of an interincisal maxillary diastema [22]. These results encourage the possibility to perform RME in young adults; this potential is greater than the orthodontist thought years ago, even knowing that currently, the MARPE technique offers interesting results in patients around 25 years [29–31]. However, would be necessary for further studies to evaluate the CBCT scans of maxilla and then predict the RME possibility retrospectively on patients who had RME treatment.

Likewise, Angieleri et al. [16] found that 12% of adult patients, the middle palatal suture was not fused. In contrast to our study, we found that individuals aged over 20 years have the possibility to find midpalatal suture opening that varies according to sex (24.5% of men and 9.6% of women). The knowledge of these data may help the orthodontist to get a more suitable explanation to offer the patient a possible treatment plan that after will be corroborated with an auxiliary examination (occlusal radiographs or CBCT). Also, this study evidences that for the RME protocol, mainly in young adults, the use of CBCT could be useful to verify the ossification status of the MPS and make the decision to perform a conventional RME (if the MPS is clearly open) or to use micro-implants (MARPE protocol) if the suture is in the process of closure, or finally, if the suture is completely closure, the SARME protocol could be the better option. However, if with other biological determinants, it is possible to establish with approximation the ossification status of the MPS of an individual, and the need for a tomographic examination should be reduced to avoid the load of ionizing radiation in the patient. Caution is necessary in this regard.

## Conclusions

The possibility to find midpalatal suture opening in post-adolescents and young adults is approximately 20%, greater than the orthodontists considered years ago. Furthermore, men are more likely to present midpalatal suture opening. These implications might be considered by the orthodontists when RME treatment is required.

Besides, the ossification of the middle palatal suture is very variable; thereby, the use of CBCT might be recommended to evaluate the midpalatal suture opening and therefore to verify the possibility of maxillary disjunction.

## Data Availability

The authors declare that the materials are available.

## Abbreviations

CBCT: Cone-beam computed tomography
MARPE: Miniscrew-assisted rapid palatal expansion technique
MPS: Midpalatal suture
RME: Rapid maxillary expansion
SARME: Surgically assisted rapid maxillary expansion
SPSS: Statistical Package for the Social Sciences
STATA: Syllabic abbreviation of the words statistics and data
